# Method to Reduce Target Motion Through Needle–Tissue Interactions

**DOI:** 10.1007/s10439-015-1329-0

**Published:** 2015-05-06

**Authors:** Matthew J. Oldfield, Alexander Leibinger, Tian En Timothy Seah, Ferdinando Rodriguez y Baena

**Affiliations:** Department of Mechanical Engineering, Imperial College London, Exhibition Road, South Kensington, London, SW7 2AZ UK

**Keywords:** Finite element method, Digital image correlation, Soft tissue, Friction, Needle insertion, Tool–tissue interaction, Biomimetic, Gelatine

## Abstract

During minimally invasive surgical procedures, it is often important to deliver needles to particular tissue volumes. Needles, when interacting with a substrate, cause deformation and target motion. To reduce reliance on compensatory intra-operative imaging, a needle design and novel delivery mechanism is proposed. Three-dimensional finite element simulations of a multi-segment needle inserted into a pre-existing crack are presented. The motion profiles of the needle segments are varied to identify methods that reduce target motion. Experiments are then performed by inserting a needle into a gelatine tissue phantom and measuring the internal target motion using digital image correlation. Simulations indicate that target motion is reduced when needle segments are stroked cyclically and utilise a small amount of retraction instead of being held stationary. Results are confirmed experimentally by statistically significant target motion reductions of more than 8% during cyclic strokes and 29% when also incorporating retraction, with the same net insertion speed. By using a multi-segment needle and taking advantage of frictional interactions on the needle surface, it is demonstrated that target motion ahead of an advancing needle can be substantially reduced.

## Introduction

The advantages of minimally invasive surgery to patients can include reduced trauma and risk of infection, improved recovery times and shortened hospital stays. Needle insertions are a common part of many minimally invasive procedures, such as brachytherapy and diagnostic biopsies. A critical part of these procedures is the ability to accurately reach targets identified pre-operatively. Biopsy needles can cause substantial displacement and deformation of tissue.^1^,^24^ The result of tissue displacement during insertion may be target motion and the consequent ineffective delivery of therapy or misdiagnosis. Intra-operative imaging can aid targeting, but with the disadvantage of hardware incompatibility in magnetic resonance imaging environments and resolution limitations when using ultrasound.

Target motion is caused by needle–tissue interactions and these interactions have been the subject of sustained investigation, particularly in the context of robotic needle delivery.^2^ Three principal mechanisms combine to cause tissue distortions and, hence, target motions: tissue displacement by the needle; tissue drag along the interface between the needle and substrate; and resistance of the substrate to the cutting action of the needle.

Large gauge needles and depth of insertion may result in significant displacements of a substrate in advance of the needle tip.^10^ As the depth of insertion increases, the influence of friction on the needle–tissue interaction becomes greater, while that of cutting remains constant. Frictional interactions have been the subject of considerable investigation in their own right.^4^,^13^ However, little research has been conducted that seeks to exploit these frictional aspects and they are often viewed as detrimental.

Biomimetics is one area where contact interactions between an invasive probe or needle have been identified as beneficial. Insertion of a mosquito proboscis and a ‘negative stiffness’ phenomenon have been identified.^3^ Two further examples of bio-inspired innovations in the area of needle design make extraction more difficult than insertion. The first, based on porcupine quills,^6^ incorporates barbs into the needle design and the second needle, using features of an endoparasite,^25^ has a tip that enlarges when coming into contact with water. A biomimetic approach, inspired by the ovipositors of wood-boring wasps, has also been used to create a steerable, multi-part needle for soft tissue surgery.^15^ Interactions between this type of needle and a gelatine tissue phantom have been investigated and visualised at high-resolution.^14^,^19^ Digital image correlation techniques offer unique capabilities to quantify the displacements and strains around a needle non-invasively and at sub-millimetre scale. Such a resolution is vital when analysing the mechanics that lead to sticking and sliding between a needle and its substrate.

There is a need to improve the accuracy of targeted needle insertions. This can be achieved using a variety of methods, including accurate modelling of anticipated tissue deformation and intra-operative imaging.^7^,^8^ Modelling, while offering potential, is computationally intensive and requires non-trivial integration of patient-specific information. Intra-operative imaging is also time consuming and imposes hardware restrictions. It is, therefore, necessary to reduce both the reliance on and frequency of imaging while maintaining or improving the targeting accuracy of diagnostic or therapeutic devices.

Using numerical and experimental methods, coupled with high-resolution digital image correlation, it is demonstrated here that some of the very interactions that cause target motion can be harnessed to the advantage of medical practitioners. The STING device consists of multiple, axially-interlocked segments,^5^ and by independently actuating each segment, it is demonstrated that static friction is used to significantly reduce the motion of a target on the needle axis. Insight offered by numerical simulations demonstrates that there are optimum actuation strategies for limiting target displacement during insertion. Experimental data demonstrates a qualitative agreement with the methods of actuation proposed. By making interactions around the internal contact interface visible, the experimental technique offers a route to optimise and tune needle actuation profiles and, therefore, minimise target displacement.

Finite element simulations are first created to test different actuation strategies for a multi-part needle. Experimental tests using the actuation strategies derived from the finite element models are then captured using digital image correlation methods. Results from both simulations and experiments are presented and discussed from the perspective of optimising the actuation strategy. Finally, conclusions relating to this work and avenues for further investigation are highlighted.

## Materials and Methods

### Finite Element Model

A finite element model was created to test whether different actuation mechanisms of a multi-segment needle could result in reduced target motion. The model, using ABAQUS finite element software, allowed different actuation strategies and the impact of varying constitutive behaviour to be studied.

**Figure 1:**
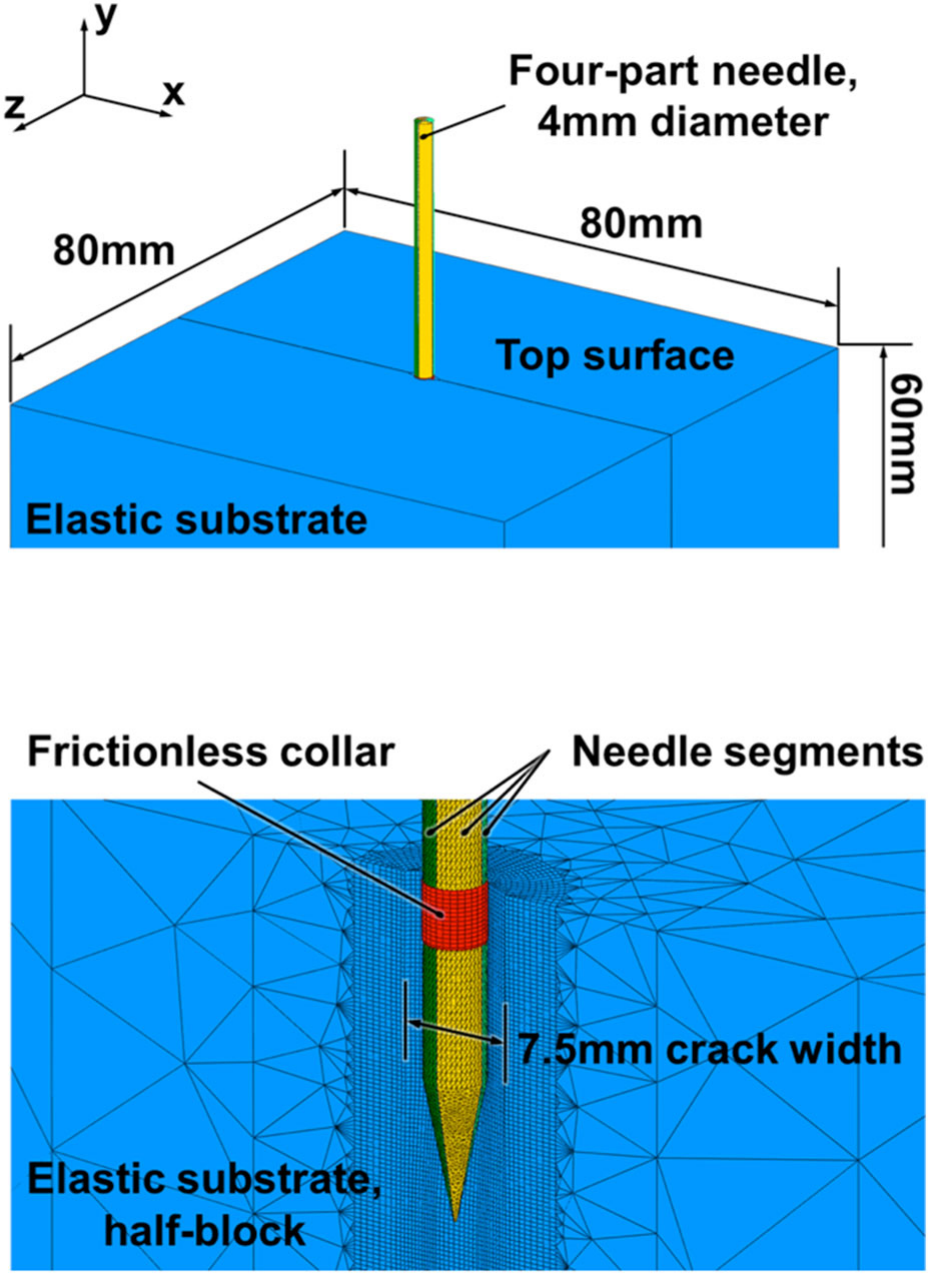
Coordinate frame and significant features of the finite element model. Top, the substrate and needle dimensions and the axially restrained top surface. Bottom, mesh and crack dimensions in a cutaway view, also illustrating the collar used to provide mesh stability on the top surface.

A continuous elastic block of 80 mm × 80 mm × 60 mm was constructed with a planar crack, 7.5 mm in width,^20^ running from the top surface to a depth of 55 mm (Fig. 1). Both faces of the crack were free and independent. This linearly elastic block, also known as the substrate, had a Young’s Modulus of 7 kPa, which corresponds to values used in previous studies^20^ and falls in the range found experimentally for soft tissues such as brain.^16^ The Poisson’s ratio in the elastic block was taken to be 0.45 to approximate incompressible conditions.

**Figure 2:**
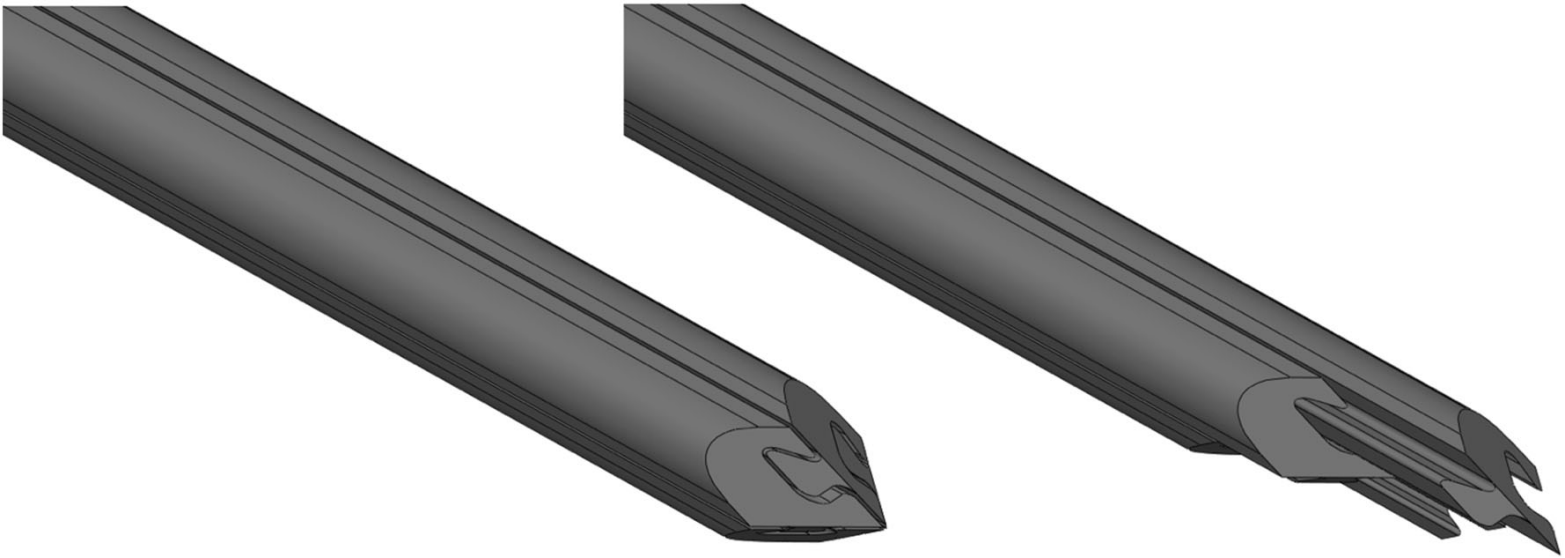
Schematic drawing showing experimental needle segments aligned in the Direct Push configuration (left) and under Reciprocal Motion with pullback actuation, also illustrating the axial interlock mechanism between segments (right).

A four-part needle, 4 mm in diameter, with a 20° included angle per segment in a conical tip, was used for the insertions. Each needle part was identical and represented a quarter of the needle when divided along the insertion axis. A schematic representation of the needle design is provided in Fig. 2, showing the axial mating between needle segments and the ability to move each segment independently. The needle was assumed to be rigid and would not deviate from its insertion axis during the simulation. A frictionless and rigid collar, 0.1 mm wider in diameter than the needle and 3.7 mm deep, was inserted into the top surface of the substrate (Fig. 1, bottom) to prevent excessive distortions and collapse of fewer than ten elements that could occur. The collar, used exclusively in the simulations and not present experimentally, interacted with the substrate by providing a small amount of separation between the substrate and needle at the insertion point. It was judged to have minimal impact on the displacements measured over the significant part of the insertion depth.

Encastré boundary conditions were applied to the edges of the substrate lying in the insertion axis. The top surface of the substrate, where the needle enters, was also prevented from moving in the insertion direction. This replicated an experimentally observed adherence of the top/insertion surface of the substrate to the wall of the substrate container, while still allowing a crack to open and the needle to enter. The opposite substrate face to the insertion surface was left unconstrained to allow as much motion of the substrate ahead of the needle insertion as possible. Coulomb friction interactions were applied between the needle segments and crack surfaces using a penalty contact algorithm. This enforced the most stable contact conditions between the rigid needle and highly deformable substrate, and allowed large relative motions between sliding surfaces. While more complex frictional interactions may be necessary to replicate conditions in, for example, poroelastic and other more complex biomaterials, previous experiments^20^ have shown a simple Coulomb model to be adequate to replicate the response of gelatine substrate to needle insertions.

Quasi-static insertions were performed in two stages using an ABAQUS Explicit solver. Firstly, stable contact conditions were set up at the top surface of the substrate block to prevent occasional collapse of substrate elements. This stability was achieved by pre-inserting the frictionless collar (Fig. 1, bottom) and all needle segments by under 15 mm. Subsequently, the four needle segments were actuated. The total length of this insertion was 40 mm and three types of actuation profile were considered:

• Direct Push (DP) Segments inserted at the same time and same rate;
• Reciprocal Motion (RM) Segments inserted sequentially with a stroke length of 4 mm in a clockwise fashion;
• Reciprocal Motion with Pullback (PB) Segments inserted sequentially with a pullback of three out of the four segments (those which remain stationary in the RM-based motion profile), specified as a percentage of the stroke length, after each insertion.

**Figure 3:**
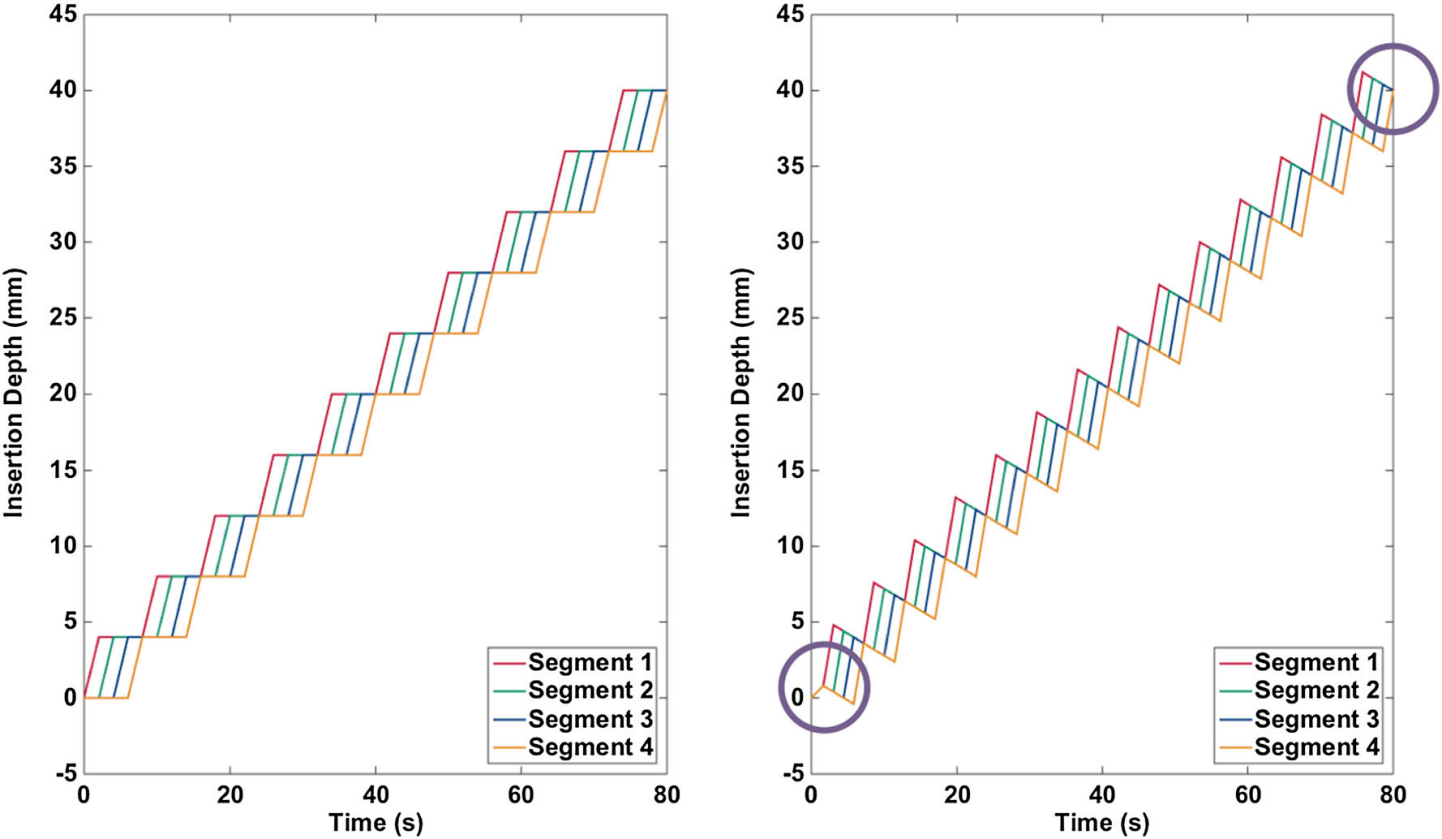
Reciprocal Motion (left) and Reciprocal Motion with 30% Pullback (right) insertion sequences giving a net insertion rate of 0.5 mm/s and segment alignment at a point 40 mm inside the gelatine substrate. The Direct Push required at the start of the motion profile and the resultant alignment of segments after 80 s (an insertion depth of 40 mm) is highlighted.

Reciprocal Motion and Reciprocal Motion with Pullback are illustrated, for the experimental cases, in Fig. 3. In all cases, the motion profiles were designed to give the same net insertion speed of 0.5 mm/s with all of the segments becoming instantaneously aligned at an insertion depth of 40 mm. A net speed of 0.5 mm/s was chosen as a compromise between a common insertion speed of 1 mm/s for Direct Push^17^ and the need to minimise increased segment velocities when using Reciprocal Motion with Pullback. The motion profiles in Fig. 3 also correspond to the actuation profiles used during modelling, with the exception that insertion velocities were scaled to save computation time. Around the crack, where displacements and strains were likely to be most significant, 8-noded, hexahedral, reduced-integration elements were used. Away from this region, 4-noded, tetrahedral elements were chosen. The maximum element dimension on the crack surface was less than 0.5 mm and a strong mesh bias to this surface enabled large elements—maximum element dimension 60 mm—to be used where strains were negligible, thus minimising the overall simulation time while retaining the necessary model resolution. Convergence tests were run to establish that the mesh density was sufficient to capture the stresses at the needle-substrate interface. Viscous damping dissipation and kinetic energy in the substrate showed the material was not too heavily damped and quasi static conditions could be assumed.

For Direct Push and Reciprocal motion, friction coefficients, *μ*, of 0, 0.1, 0.2 and 0.3 were tested. When considering Reciprocal Motion with Pullback, *μ* = 0.2 was used. Previous investigations suggest higher values for contact between needles and dry gelatine. However, *μ* = 0.2 was the largest value that consistently avoided instabilities for all simulated actuation profiles. Pullback percentages of 5, 10, 15, 20, 25, and 30% were then implemented.

Displacements of nodes on the crack surface 20.6 mm before the crack termination and of nodes 1 mm ahead of the planar crack termination, in its plane, across a width of 4 mm, were recorded. The strain energy developed in the whole substrate was also monitored to provide an indication of whether the insertion methods were likely to be tissue sparing.

### Experimental Method

A physical experiment was performed to test the findings provided by the finite element model. The four-part needle of the same dimensions was made of Vero Black (Stratasys, Minnesota, US), which was rigid compared to the substrate. To achieve independent motion, the needle was divided into four axially interlocking segments, with each actuated separately from the rear. The tip of the needle was a flat bevel tip, with a 20° included angle per segment (Fig. 2).

To consider the actuation strategies from the finite element studies, three cases were considered experimentally:

- Direct Push with an insertion rate of 0.5 mm/s;
- Reciprocal Motion with a stroke length of 4 mm, equivalent to a net insertion velocity of 0.5 mm/s and maximum segment speed of 2 mm/s (Fig. 3);
- Reciprocal Motion with Pullback of 30% and a 4 mm stroke length, as this was suggested as having the strongest effect in the numerical cases (Fig. 3). The net insertion velocity was, again, 0.5 mm/s with a maximum segment speed of 2.9 mm/s.

Gelatine was used as the experimental substrate due to its transparency, availability and controllable stiffness. Here, 3.5% by mass of bovine gelatine (Sleaford Quality Foods, Sleaford, UK) was added to warm water and dissolved. The mixture was poured into containers with an 80 mm × 80 mm cross section to a depth of 78.5 mm. For the purpose of performing digital image correlation, 1 g of aluminium oxide (Al_2_O_3_) particles less than 10 μm in diameter was added to the gelatine solution. The mixture was set in a fridge overnight and, subsequently, allowed to reach room temperature, 21 ± 2 °C, before performing needle insertions.

A 4.5 mW, 532 nm, DPSS, collimated laser (Thorlabs Ltd., Ely, UK), in conjunction with a series of lenses, generated a light sheet in the gelatine tissue.^21^ This light sheet was scattered by the suspended Al_2_O_3_ particles and the signal was recorded by a CCD camera (AVT Guppy Pro F-146B, Stadtrod, Germany).

**Figure 4:**
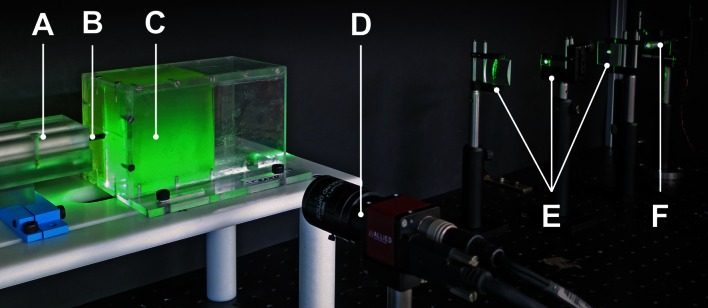
Experimental equipment: (a) trocar to deliver needle; (b) four-part needle; (c) gelatine substrate illuminated by the laser light sheet; (d) CCD camera; (e) three lenses used to generate a planar light sheet; (f) laser illumination.

Individual needle segments were driven by linear actuators, powered by Maxon A-max 22 DC motors (Maxon Motors, Sachseln, Switzerland) and controlled through a compactRIO embedded controller programmed in LabView (National Instruments Inc., Austin, USA). The insertion axis and vertical light sheet were aligned with the centre of the box cross section to minimise edge effects. Figure 4 shows the principal components of the experimental setup.

Regardless of the method used for actuation, the tips of the four needle segments were brought together at a point that fell within the 16.4 mm × 12.3 mm field of view that was centred on the insertion axis and began 35.6 mm from the insertion point.

To verify the numerically observed behaviour, the axial displacement of points across the needle diameter and 1 mm in front of the point at which the four needle segments became aligned at the end of the insertion, were monitored. This enabled the target motion for the different actuation strategies to be compared when the needle had reached the equivalent configuration after the same amount of time. A minimum of fourteen insertions was performed for each actuation method, with each insertion in a fresh, undamaged block of gelatine.

## Results

The first set of results to be analysed were those from the finite element simulations. Due to the large number of repetitions required experimentally, the simulations enabled the Reciprocating Motion with Pullback actuation strategy that was likely to have the most significant effect to be identified. Limitations of space dictate that only those simulations where *µ* = 0.2 are shown here. In the cases of Direct Push and Reciprocal Motion, the axial displacements at both the monitored nodes on the crack surface, and those monitored 1 mm ahead of the crack termination, increased with the friction coefficient.

**Figure 5:**
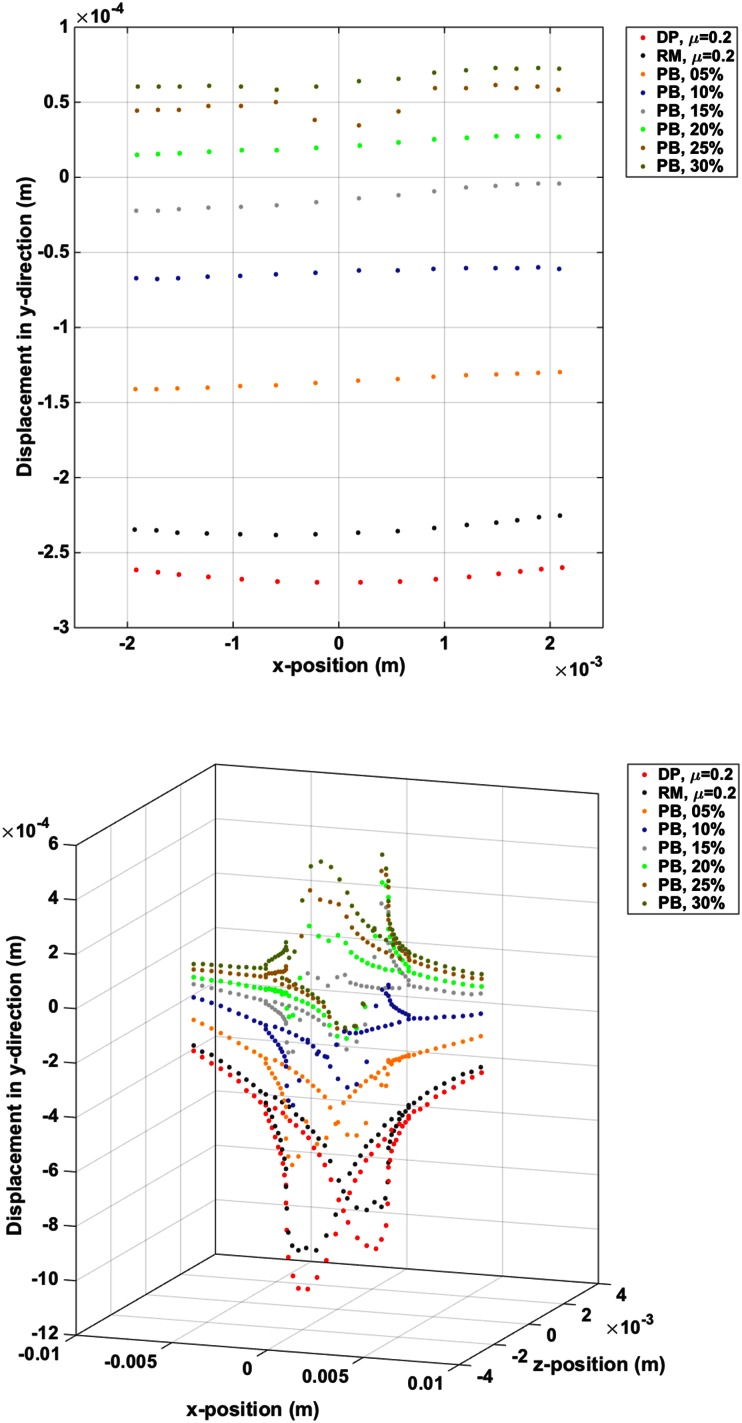
Top, displacements 1 mm ahead of the crack tip that were essentially planar in nature and showing the benefit of Reciprocal Motion with Pullback. Bottom, displacements around the planar crack, at the end of the simulated insertion, showing the crack conforming to the needle and reduced overall drag when using Reciprocal Motion and Reciprocal Motion with Pullback.

Figure 5(top) shows the nodal displacements across the diameter of the needle, 1 mm ahead of the crack termination. The largest target motion is seen with Direct Push actuation, followed by Reciprocal Motion. When including Pullback, the amount of target motion further decreases. As Pullback increases, the amount of target motion decreases in all cases. Though the displacements are themselves small, the Reciprocal Motion with 30% Pullback shows less than 50% of the target motion for Direct Push.

Similar trends are seen for the nodes monitored along the crack surface. The profile of the planar crack as it is opened by the passage of the four-part needle can be seen in Fig. 5(bottom). Displacement magnitudes in the axial direction are much greater than in the nodes monitored ahead of the crack termination. Again, increasing amounts of pullback reduce the amount of axial displacement in the direction of insertion. In Fig. 5, the Reciprocal Motion renders the displacement profile asymmetric suggesting that stick–slip behaviour and the order of segment actuation play a role in the overall substrate deformation. It is also apparent that, despite the advantages of Reciprocal Motion with and without Pullback, the incremental benefits of Pullback decrease as it becomes a greater proportion of the stroke length.

**Figure 6:**
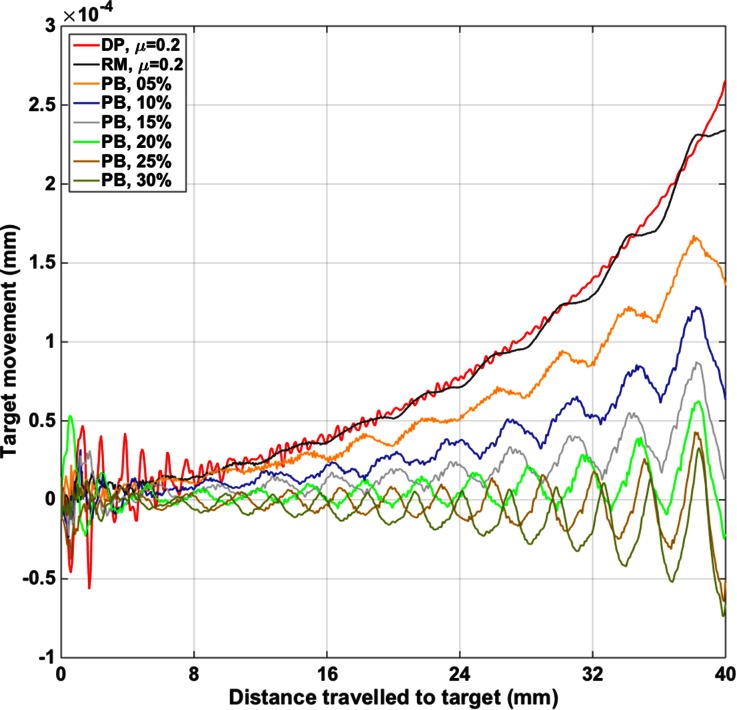
Vertical displacement at the target location over the duration of insertions. Reciprocal cycles are evident and the Direct Push insertions largely bound all other cases with a maximum deflection.

Figure 6 illustrates the movement of the target position over time. The displacements of the line of nodes 1 mm ahead of the final insertion point and across the needle diameter were averaged and then smoothed using a moving average filter. The Direct Push response forms a bound on the axial displacement (*y*-direction) of the target nodes except for occasional Reciprocal Motion transgressions. Reciprocal Motion with Pullback is most effective at reducing the target motion and even drags the material back towards the needle insertion point when pullback is 20% or greater.

To identify the impact of the actuation profiles on the strain in the substrate, the overall accumulations of strain energy, at the end of the insertion are presented in Table 1. Strain energy was acquired as a standard output parameter for the whole substrate throughout the simulation. There is little difference between each actuation profile. However, the performance of the Reciprocating Motion with Pullback cases is influenced by the small amount of calibrating direct push (Fig. 3right) at the beginning of the insertion. This short period of direct push motion ensured that the probe segments became aligned at an insertion depth of 40 mm for all actuation profiles. The calibrating direct push was applied at the beginning of the insertion where its influence on the motion of the target would be minimised (Fig. 6).

**Table 1:** Accumulated strain energy at the end of the simulated insertions.

	DP *µ* = 0.2	RM *µ* = 0.2	PB 5%	PB 10%	PB 15%	PB 20%	PB 25%	PB 30%
Strain energy (mJ)	1.02	1.01	0.96	0.94	0.94	0.94	0.94	0.95

This can be seen when the strain energy is plotted against time, but is not included here due to the limitations of space. The small amount of direct push relatively increases the amount of accumulated strain energy at the beginning of the insertions. It can, therefore, be assumed that Reciprocal Motion with Pullback still offers improved performance—less accumulated strain energy—than Direct Push and Reciprocal Motion alone.

**Figure 7:**
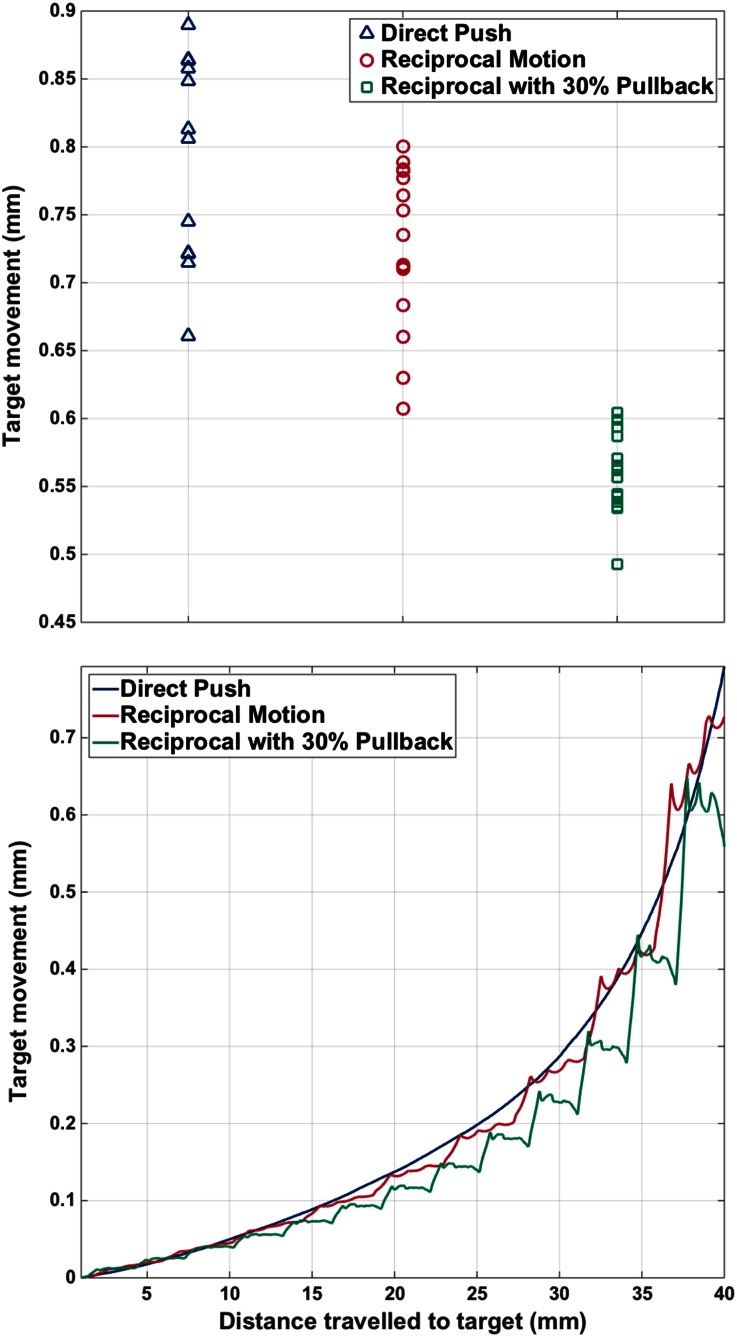
Top, target displacements ahead of the needle at the end of the experimental insertions. Bottom, target motions averaged over equivalent insertion points for each actuation strategy.

Experimentally, the vectors measured across the diameter of the needle, 1 mm in front of the point where all needle segments finally align in the field of view, were averaged at the end of insertions in Fig. 7(top). A point 1 mm in front of the needle alignment position matches that of the simulations and is insensitive to imaging artefacts, such as the reflection of light from the needle tip. Target displacements seen experimentally are greater for all actuation profiles than those seen in the simulations, with the component along the insertion axis dominating.^19^

Quantification of the relationship between actuation mechanisms is done by comparing the target motions at the end of each insertion. Each target motion is characterised by averaging the displacement of measurement points, across the needle width, 1 mm in front of the location at which the needle segments finally align. The overall mean target motions for Direct Push, Reciprocal Motion and Reciprocal Motion with Pullback are 0.79, 0.73 and 0.56 mm respectively. Using a *p* value of 0.05 and Welch’s *t* test there is statistically significant difference between all combinations of actuation mechanisms.

The time-averaged target displacement for all of the needle insertions is shown in Fig. 7(bottom). Each actuation profile shows a non-linear increase in displacement as the needle approaches the measurement point. Both Reciprocating Motion and Reciprocating Motion with Pullback result in less target motion than the Direct Push equivalent, until the target is neared. The final target motion is, however, also below the bounding displacement of the Direct Push actuation when all probe segments come to rest in alignment.

## Discussion

By considering the displacement of material in front of the needle, a situation analogous to target motion is investigated. Here, it is hypothesised that the contact properties between a novel, multi-segment needle and substrate can be used to reduce the displacement of the material in front of the needle and, hence, reduce target motion in clinical applications. Results in Figs. 4, 5, and 6 demonstrate that a multi-segment needle can, when the segments are actuated independently, diminish the magnitude of target motion when comparing the equivalent net insertion.

Initial finite element models indicate that a four-part needle offers significant benefits in reducing target motion and moderate benefits in reducing the overall amount of strain in the surrounding substrate. Table 1 indicates that the small benefit to the overall strain in the substrate by using Reciprocal Motion with Pullback is quite consistent among all of the different pullback amplitudes tested. Small amounts of Direct Push at the beginning of the actuation profiles had a disproportionate impact on the overall profile of the accumulated strain energy. Modifying the profile to eliminate the requirement for direct push, by using a short, initial Reciprocal Motion with Pullback loop, may improve performance even further. It should be noted, however, that the overall strain energy does not account for local peaks in strains. Particularly in the case of shear strain around the edges of the needle segments, these can be greater for transient periods than in the cases of Direct Push and Reciprocal Motion. Local peaks in strains during sticking and sliding result in cyclic profiles in the strain energy when reciprocal motion of any kind is present. These cyclic profiles are imposed on the more general trend of reducing overall strain energy compared to Direct Push over the course of insertions. Complicated interactions between strains and stick–slip contacts require careful tuning to optimise performance and it may not be possible to optimise both target motion and strain profiles imparted on the substrate.

Frictional properties, notably sticking contact, enable the substrate to be anchored by the unmoving needle segments. The small amount of retraction gradually pulls the substrate back towards its undeformed configuration after it has been dragged forward during insertion. Similarly, the continuity of the tissue around the needle means that there is sticking ‘anchor’ of stationary substrate on either side of the portion of material that is being dragged by the forward-moving needle segment.

The complexity and significance of contact interactions also indicate the importance of using the correct interaction behaviour in the model. Friction models incorporating elastic and hysteretic effects are more likely to better capture the type of response seen in a broad range of phantoms and *ex vivo* tissue.^4^ The simulations presented here are, therefore, a strong indicator of the practical benefits of the respective actuation profiles, but are not definitively validated. Another weakness of the finite element model was the lack of cutting that advances with the needle insertion. Pre-existing cracks offer a reasonable assumption, but many needle insertion papers also demonstrate the significance of cutting effects.^12^,^18^ These can be anticipated to provide additional axial displacement of the substrate both around the shaft and ahead of the tip. The net backward motion of the target due to the lack of cutting processes, seen in Figs. 5 and 6, is an exaggeration of what is possible experimentally (Fig. 7).

Despite the limitations of the finite element model, sufficient behaviour is observable to merit testing the different actuation mechanisms experimentally. A pullback of 30% was chosen as, when modelled, it provided the least target motion, substrate displacement on the crack surface and a small imparted strain energy, once the needle cycled to its final position.

Though not investigated here, the order in which the segments are actuated may also cause the pullback method to be more or less effective. Segments were actuated in the sequence clockwise 1–2–3–4. However, the alternative 1–3–2–4 sequence may, in the future, prove more effective. Similarly, if the orientation of the needle segments in relation to a planar crack is rotated through 45°, the ability of the segments to anchor neighbouring material is decreased as less of the substrate is in contact with the stationary segments. It has also been shown^12^,^23^ that the failure mode—ring-shaped, planar, triangular—is highly dependent on the shape and configuration of the needle tip.

The identified benefits of anchoring and withdrawing substrate in contact with the needle surface could be further exploited. By increasing the number of needle segments, the amount of tissue that is stationary in proportion to that being dragged could be increased. There are limitations to the effectiveness of increasing the number of needle segments as the manufacturing complexity, already significant, will become unfeasible. An additional way to improve the traction of the anchoring segments would be to provide directional texturing on the needle surface. There is a biomimetic precedence for this that has been explored.^9^,^11^,^22^ While the ability to weight the contact mechanics toward greater traction is attractive, the risk of increased tissue trauma when extracting the needle may grow prohibitively. It is noted, also, that a needle with a 4 mm outside diameter is greater than even the standard gauges used in biopsy. The principal of cooperative motion is equally applicable to smaller diameter needles.

The ability to reduce target motion will enable more accurate control and delivery of therapy or targeting of tissue, e.g., biopsy. There remains the scope to reduce the remaining, significant target motion by tuning the actuation strategy further. In Fig. 7(bottom) it can be seen that, even with pullback, the experimental target motion increases rapidly as the needle approaches. By asymptotically reducing the amplitude of the segment motion as the target is approached, it is possible that the motion will be reduced further. All reduction is beneficial, as it would decrease the required frequency of, for example, intra-operative imaging.

Digital image correlation has been shown to be an effective, non-invasive and high-resolution method for quantifying internal displacements of a tissue phantom. However, it has been observed that strain measurements can be particularly noisy due to the local derivatives required in their calculation.^19^ In this investigation, experimental strain energy data is less conclusive than displacement data and its application as a validation of behaviour seen in the numerical models is neglected. The use of planar measurements of substrate motion also has limitations. Due to the inherently asymmetric nature of reciprocal motion, measurements may suffer from motion of material across the plane itself. Assuming a planar crack as the failure mechanism of the gelatine tissue phantom, the alignment of the crack and laser light planes will influence the output data. Obtaining statistically significant output data in the experiment contributes to attenuating the implications of these effects.

## Conclusions and Future Work

Using a multi-segment needle, an actuation method is presented that reduces target motion when compared with the normal, Direct Push, of a conventional single-part device. Experimentally, when compared to Direct Push insertion, Reciprocal Insertion achieves a statistically significant 8% reduction in target motion. When applying Reciprocal Motion with Pullback of 30%, target motion is reduced by a statistically significant 29% compared to Direct Push. Simulations indicate that these reductions in target motion are not costly in terms of overall tissue strain and that it may be possible to tune the actuation specifically to reduce strain energy in the substrate.

Although the current reductions in target motion are significant, there is scope to further improve performance. Three areas have been identified for further investigation. The first is the actuation strategies and would incorporate different sequences of segment actuation, non-linear displacement profiles and displacement profiles that are optimised with the likely complex contact mechanics and relationships between the needle segment and substrate. Secondly, there is considerable scope for investigating the impact of a much wider range of complex contact interactions that are likely to be seen during needle insertions into tissues and other biomaterials. Thirdly, taking inspiration from biology, unidirectional coatings on the needle surface would accentuate the difference between insertion and retraction contact mechanics. As with all medically orientated investigations conducted in a soft tissue phantom, it is envisaged that the needle and actuation will be tested in biological, heterogeneous tissue.
